# The Gilding-the-Lily Effect: Exploratory Behavior Energized by Curiosity

**DOI:** 10.3389/fpsyg.2020.01381

**Published:** 2020-07-03

**Authors:** Mowei Shen, Pengpeng Liu, Xinyu Li, Jifan Zhou, Hui Chen

**Affiliations:** ^1^Department of Psychology and Behavioral Sciences, Zhejiang University, Hangzhou, China; ^2^Department of Psychology, Zhejiang Normal University, Jinhua, China

**Keywords:** gild the lily, superfluous behavior, curiosity, interest, deprivation, exploration

## Abstract

The widespread metaphor “to gild the lily” suggests that people usually engage in superfluous behaviors. Understanding the cognitive mechanism underlying superfluous behaviors helps individuals to reduce possible waste and even disasters incurred by unnecessary actions. Here, we assumed that curiosity for new information partly pushes people to make needless efforts. This hypothesis was tested through three experiments. In three experiments, we found that when participants knew that expending more efforts than task requirements brought no better results, they still exerted various exploratory activities to fulfill curiosity. These results imply that the impulsion to satisfy the desire for information could partly drive individuals to indulge in unnecessary activities over mission demands. Present research improves the comprehension of irrational superfluous behavior and provides directions to reduce loss and waste caused by gilding the lily.

## Introduction

People often engage in activities superfluous to the requirements of a task or mission, which have the potential to induce disasters, instead of being beneficial. For example, in January 2012, 32 passengers were killed in the *Costa Concordia* cruise ship disaster in Tuscany, Italy. Later, investigations showed that the captain’s additional manual change in the route was a major cause of the accident. The ship’s navigation system had planned a safe route for the voyage, which kept the ship away from rocks, and additional manual adjustment was not required.However, the captain took control of the ship and sailed away from the planned route. The new route was too close to the shore, and the ship eventually struck some rocks (Levs, 2012). The captain’s behavior could be described by the proverb, “to gild the lily,” which is defined as making superfluous additions to what is already complete (American Heritage, 2011), or engaging in unnecessary and usually wasteful activities (Price, 2011). Because automated technologies are extensively employed, needless intervention in human-automation interaction systems potentially entail grave losses or catastrophes (Hoff and Bashir, 2015).

Current research refers to the phenomenon of immersing in superfluous behaviors as the gilding-the-lily effect (abbreviated as GL effect), and it is usually regarded as irrational (Pielstick, 1970; Lidstone, 1995). The rationale behind gilding the lily being regarded as superfluous is twofold. First, given that gilding the lily adds no more beauty to lilies, it is waste of gold foil. Second, redundant gold probably undermines the lily’s natural appearance. In the *Costa Concordia* tragedy, since that a safe route had already been provided by the navigation system, additional manual control not only guaranteed no more safety but also might cause danger. Thus, the disaster could be interpreted as a consequence of the GL effect. Both the irrationality of the GL effect and its potential harmful effects prompted us to examine the cognitive mechanism that underlies the GL effect.

Some accounts have been proposed to explain the GL effect. The account of boredom suggests that superfluous behaviors may derive from an individual’s tendencies to dispel boredom, which is perceived to be torturous (Koerth-Baker, 2016). Koerth-Baker (2016) found in their study that when the participants were told to wait for a while, with nothing to do, and they had the chance to receive electric shocks, participants chose to give themselves an electric shock. Receiving painful electric shocks was superfluous, since the task did not require the participants to do this. Koerth-Baker (2016) suggested that when left with nothing to do, participants felt bored and tortured, and it was the impulsion to dispel boredom that induced participants to get an electric shock.

Another account that has been proposed to explain the GL effect is the account of curiosity. Curiosity is defined as the desire for new information, knowledge, experiences, or sensory stimulation, and it motivates exploratory behavior to resolve uncertainty or experience the unknown (Berlyne, 1978; Loewenstein, 1994; Spielberger and Starr, 1994; Litman, 2005; Grossnickle, 2016). When curiosity is aroused, individuals often soak themselves in impulsive activities to seek information (Arkes and Ayton, 1999; Shani and Zeelenberg, 2007; Kruger and Evans, 2009; Barkan et al., 2016; Hsee and Ruan, 2016). The curiosity account suggests that, to satisfy their curiosity, humans possibly tend to perform additional actions over the task requirements (Loewenstein, 1994, 2006; Kruger and Evans, 2009; Hsee and Ruan, 2016). Specifically, Hsee and Ruan (2016) designed an experiment in which pictures of disgusting insects were projected on a screen. The participants did not know the specific content of each picture, and they could simply press keys to skip pictures in order to complete the task (Hsee and Ruan, 2016). However, the researchers found that participants still chose to view the pictures, even though they knew that the pictures were disgusting (Hsee and Ruan, 2016). Viewing these pictures was unnecessary for the task, and these superfluous behaviors were attributed to eliminating the uncertainty caused, and curiosity aroused, by the pictures left unseen. Overall, both these accounts offer explanations for the motivation to engage in needless activities.

Traditional curiosity theories and empirical evidence distinguish between two types of curiosity: the interest type and the deprivation type (Berlyne, 1978; Litman, 2005; Litman et al., 2010; Mussel, 2010). The interest type, or I-type curiosity, is induced by opportunities to gain information and is associated with acquiring knowledge simply for the intrinsic joy associated with it (Litman, 2008; Litman et al., 2010; Chang and Shih, 2019). The deprivation curiosity, or D-type curiosity, is stimulated when humans lack a specific piece of information, which is relevant to the reduction of undesirable uncertainty (Loewenstein, 1994; Litman, 2008; Litman et al., 2010; Chang and Shih, 2019). I-type curiosity is usually accompanied with positive emotions and diverse exploratory behavior, and D-type curiosity is accompanied with negative feelings and specific exploration for the missing information (Litman and Spielberger, 2003; Litman, 2008, 2010). Previous research mainly focused on the driving force of D-type curiosity. For instance, participants in the above-mentioned study did not have the information about the content of the pictures, and thus, they chose to view the photos to eliminate that aspect of uncertainty (Hsee and Ruan, 2016). In such a situation, the motivation is understood to be the D-type curiosity, which partly drove participants to make impulsive exploring acts. I-type curiosity mostly emerges in fresh and novel situations, without obvious specific uncertainty, and pushes individuals to seek possible information (Litman, 2008; Grossnickle, 2016). It remains to be examined whether I-type curiosity can stimulate superfluous behavior.

The present study had two aims. First, to examine the possibility that I-type curiosity energizes the GL effect. Previous studies on the subject usually studied the notion of specific uncertainty and showed that D-type curiosity plays an important role in the GL effect (Kruger and Evans, 2009; Hsee and Ruan, 2016). I-type curiosity differs from D-type curiosity in many aspects, and the outcomes that result from D-type curiosity cannot be directly applied to I-type curiosity (Litman et al., 2005, 2010; Litman, 2008). Thus, investigating the role of I-type curiosity in inducing the GL effect helps to enhance the comprehension of the GL effect and could potentially guide individuals to mitigate the possible losses triggered by GL effect. The second aim of the study was to compare the influence of I-type curiosity and D-type curiosity on exploratory activities. Previous research has found that D-type curiosity possibly has a more sustainable impetus for exploratory behavior (Litman et al., 2005). However, the two types of curiosity were measured as traits by the questionnaires, and the conclusion was inferred from a multiple correlation analysis (Litman et al., 2005). The facets of curiosity as a trait and a state refer to the different properties of curiosity. Trait facets reflect the frequency with which individuals experience curiosity, while state facets refer to the intensity of the curiosity experienced at a certain time, as an emotional and motivational state (Spielberger and Starr, 1994; Collins et al., 2004). In the present study, we tried to separately evoke the two types of state curiosities in the same situation and compared their effect on exploratory behavior. Thus, we could directly test the theorical assumption that D-type curiosity drives exploratory behavior more acutely (Litman, 2008; Litman et al., 2010).

We examined and compared the impact of I-type curiosity and D-type curiosity through three studies. Each study comprised of two different conditions. One was the *unknown* condition where specific uncertainty was created. The other was the *known* condition where participants were given key information about the task. This was done because the I-type curiosity is usually involved in fresh and novel situations without obvious specific uncertainty, and the D-type is often involved in situations with a specific information gap (Litman, 2008; Grossnickle, 2016; Chang and Shih, 2019). We inferred that I-type curiosity was mainly related to the *known* condition, and D-type curiosity was more concerned with the *unknown* condition. Thus, we could identify the exploring activities in the *known* condition and contrast it with behaviors in the *unknown* condition.

## Study 1

Study 1 simulated a scene where a spaceship and space station were docked. Participants played the role of astronauts to monitor a spaceship that was going to dock at a space station. Before docking, they should make sure that docking requirements were fulfilled. If the automatic system on the spaceship failed to accomplish preparatory work, participants needed to manually finish docking preparation. Half of the participants were assigned to the *known* condition, in which they were clearly informed that, when preparatory work for docking was done, extra manipulation no longer helped to improve the success rate of docking. The other half of the participants were assigned to the *unknown* condition, in which they were not aware of whether extra manipulation would be useful. In both conditions, requirements for docking would always be finished by the automatic system, and we examined whether participants would still sail the spaceship manually.

The information gap in the *unknown* condition was expected to activate D-type curiosity, because participants lacked the information to understand whether shrinking deviation could further improve the docking success rate. In the *known* condition, such an uncertainty was removed; therefore, I-type curiosity was expected to emerge to impel participants to search for any new information in the scenario. Due to the influence of D-type and I-type curiosities, we expected that participants would make extra manipulations in both conditions. And given that D-type curiosity possibly exhibits a larger effect on exploration than I-type curiosity, the proportion of participants who manually controlled the spaceship would be higher in the *unknown* condition. All measures, manipulations, and exclusions in Study 1 are reported below. No more data were collected contingent on initial analysis.

### Materials and Methods

#### Participants

Based on the results of the pilot studies, we predicted an effect size of *w* = 0.34 for our experimental design. We performed a power analysis with G^∗^power 3 (Faul et al., 2007), which determined that at an α level of 0.05, 69 participants were needed to detect an effect with a power of 0.80.Sixty-nine participants were finally recruited: 34 in the *unknown* condition [19 women; mean age (M_age_) = 23.35, standard deviation (SD) = 2.40; age range = 19–30 years] and 35 in the *known* condition(21 women; M_age_ = 22.89, SD = 2.24; age range = 20–27 years). Participants were paid to take part in the experiment and signed consent forms. All participants had normal or corrected-to-normal visual acuity. The study was approved by the Research Ethics Board at the Department of Psychology and Behavioral Sciences, Zhejiang University.

#### Procedure and Design

**FIGURE 1:**
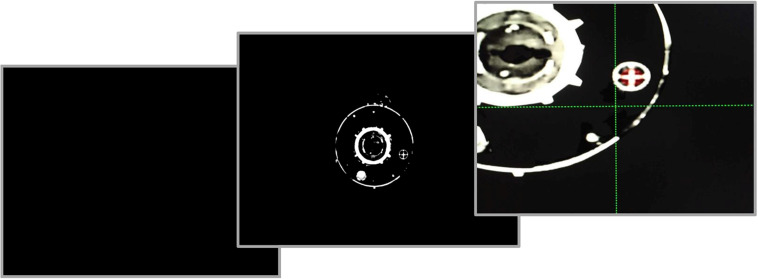
The scene that participants saw on the monitor. When the spaceship was far from the space station, they saw nothing but darkness. When the spaceship gradually caught up with the space station, participants could see its outline. When the spaceship was near enough for docking, participants could see the mark for docking clearly. The dashed green cross is the location mark of the spaceship, and the white cross is the mark of the space station. When the intersection of the green cross was in the red circle within the white cross, the position deviation between those two aircrafts met the requirement for docking. The radius of the red circle was 23 pixels.

The participants were tested in a dark room individually. All displays were presented on a Cathode Ray Tube (CRT) monitor of a 17-inch computer. Participants were informed that they would take part in an ergonomics study in which they played the role of an astronaut. A spaceship was going to dock at a space station under the control of an automatic system. The mission for the astronaut was to monitor whether the automatic system had successfully prepared for docking. If the automatic system failed to prepare per the standards, the astronaut needed to make manual corrections. In the first part of experiment, the spaceship sped up to reach the space station. Participants gradually saw the space station on the screen. When the spaceship caught up with space station, the second part began. During the second part, two systems worked together to adjust the position deviation between the spaceship and the space station: the self-calibration system of the spaceship and the manual system controlled by astronaut. The self-calibration system worked first and then came the manual system. Before the adjustment period ended, participants had to ensure that the position deviation between the spaceship and the space station was less than a required threshold (Figure 1).

Half of the participants were assigned to the *unknown* condition arbitrarily, and they were informed that once the green cross center was in the red circle, the spaceship could dock at the space station. The other half participants in the *known* condition were further informed that once the position deviation meets the docking requirement, any additional manual control would not change the success rate of docking. In both conditions, before the manual operation phase, the self-calibration system would successfully adjust the green cross center into the red circle. Specifically, the intersection finally randomly lay in a concentric circle within the red circle. The radius of this concentric circle was 15 pixels. Therefore, in both conditions, the qualification for docking was met. This experiment took about 5 min. Before the experiment, each participant would view an accelerated docking process and manually control the spaceship to reduce uncertainties about the course and operation of study. After the simulation task, participants received a questionnaire about their understanding of the task and the reason they chose (or not) to take extra control. Those questions were mixed with other filler questions to collect participants’ subjective experiences with the docking system.

### Analysis and Results

In the *unknown* condition, two participants who reported that the self-calibration system had not adjusted the cross center into the red circle were excluded; in the *known* condition, three participants were excluded for the same reason. Consequently, the data from 64 participants (32 in each condition) were analyzed. We used SPSS 20.0 for data processing.

In the *unknown* condition, 27 participants manually controlled the spaceship, and 5 participants gave up the chance of manipulation. The number of participants who manually controlled the spaceship was significantly higher than those who did not control the spaceship [χ^2^(1) = 15.13, *p* < 0.001, *w* = 0.49]. In the *known* condition, 31 participants manually controlled the spaceship, and 1 participant gave up the chance to manipulate the spaceship. The number of participants who manually controlled the spaceship was also significantly higher than the number of participants who did not control [χ^2^(1) = 28.13, *p* < 0.001, *w* = 0.66, respectively]. However, the proportion of participants who chose to control the spaceship in the *unknown* condition was not significantly higher than the proportion in the *known* condition [χ^2^(1) = 1.66, *p* > 0.05,*w* = 0.16].

In the *unknown* condition, among the five participants who did not control the spaceship, four participants thought that work had been done by the automatic system, and there was no need for manual control; one participant reported that the automatic system was more precise, and manual manipulation might lead to a bigger deviation. Of the 27 participants who manually manipulated, one participant stated that she/he just needed to do something, one felt obsessive if he/she gave up, and 25 participants reported that they wanted to improve success rate. In the *known* condition, all 32 participants reported that the automatic system had completed preparatory work for docking, and extra efforts did not help to improve success rate of mission. The participant who gave up manipulating the spaceship stated that the docking requirements were already achieved and there was no need for further corrections. Among the remaining 31 participants, 3 participants wanted to have a try, 12 participants clearly reported that they felt obsessive if they gave up manipulating, and 16 participants stated that they shrunk the deviation because they wanted to have a try to improve the success rate.

### Discussion

In the *unknown* condition, the D-type curiosity drove participants to control the spaceship. We also detected significant human intervention in the *known* condition. When participants explicitly knew that extra manipulation brought no additional benefits, they still shrank the position deviation. The GL effect in the *known* condition indicated that I-type curiosity partly pushed participants to indulge in unnecessary behaviors beyond task requirements. However, we detected no significant differences between the *unknown* and *known* conditions. This could partly be because there was only one test without feedbacks in Study 1. Participants had no information to revise their exploratory behavior. Both the force of D-type and I-type curiosities possibly reached the ceiling. Therefore, more trials might be needed to exhibit the intensity and persistence of D-type in the *unknown* condition. Despite the absence of the manipulation difference between the *unknown* and *known* conditions, we detected different levels of goal diversities in each condition. In the *unknown* condition, most participants reported that they wanted to improve the success rate. In the *known* condition, participants’ reasons for manipulating the spaceship were more diverse. This is consistent with findings that D-type curiosity involves a desire for success, and I-type curiosity is associated with various exploration (Litman, 2008).

It is also worth noting that some subjects reported being obsessive if they gave up controlling the spaceship. The results of questionnaire showed that participants fully understood that manual manipulation did not help improve the success rate when docking requirements were fulfilled by the automatic system, and they did not know the exact reason why they felt obsessive and why they chose to manually control the spaceship when the preparation had been done. Such reports of feeling obsessive might be the rational *post hoc* attribution of superfluous behaviors in the current study. Considering that subjective reports were vague and lagging and in all three studies, we mainly focused on the analysis of behaviors during the task. To further test the effectiveness of I-type curiosity and compare its intensity with D-type curiosity, we designed a multiple-trial situation with feedbacks in Study 2.

## Study 2

The design of Study 2 was abstracted from popular mobile role-playing games such as “Onmyoji.” In such games, players usually need to draw summon circles to send for different roles and spend lots of resources to cultivate those roles. We were inspired by players’ extra behaviors during games. The probability for summoning different roles is actually constant and unrelated with the type of summon circles. However, we have observed that players often rack their brains for various summon circles to send for desired roles. Similar behaviors are frequently seen in daily life. For example, some people usually blow on the dice before they roll them, even when they know that blowing does not have any specific benefit because the outcome is randomly generated. In Study 2, we simulated the summoning mechanism and designed a drawing game. We manipulated the existence of specific uncertainty and analyzed explorative activities in the *unknown* and *known* conditions. All measures, manipulations, and exclusions in Study 2 are subsequently reported. No more data were collected contingent on initial analysis.

### Materials and Methods

#### Participants

Based on the results of our pilot studies, we predicted an effect size of Cohen’s *d* = 0.69 for our experimental design. To ensure adequate power (0.80), we performed a power analysis with G^∗^power 3 (Faul et al., 2007), which determined that when α = 0.05, we required a sample size of ~67. Sixty-seven participants were finally recruited: 33 in the *unknown* condition (16 women; M_age_ = 21.09 years, SD = 2.43; age range = 18–28 years) and 34 in the *known* condition (25 women; M_age_ = 20.84 years, SD = 2.79; age range = 18–28 years). Participants were paid to participate in the experiment and signed consent forms. All participants had normal or corrected-to-normal visual acuity. The study was approved by the Research Ethics Board at the Department of Psychology and Behavioral Sciences, Zhejiang University.

#### Procedure and Design

**FIGURE 2:**
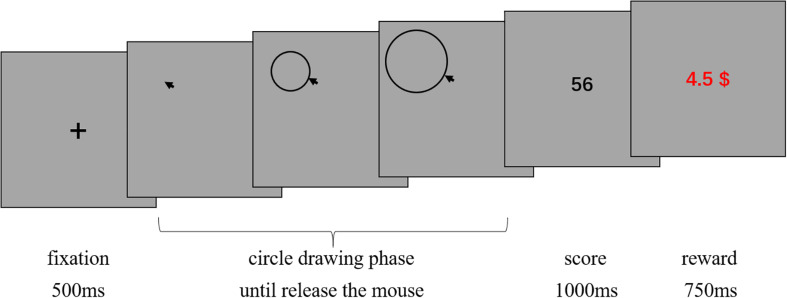
The procedure used in Study 2. For each trial, a fixation cross appeared on the screen for 500 ms; participants were required to draw a circle on the screen by continually pressing the mouse button and dragging the mouse. The center of the circle was located where the mouse button was pressed. The circle enlarged as the mouse was dragged away from the initial location, or shrank when the mouse was dragged toward the initial location. When the mouse button was released, the drawing of the circle was finished. The radius was the distance between the pointer and the center. When the circle drawing phase ended, the score they received was presented for 1 s. Then, the reward amount appeared on the screen for 750 ms. The reward was calculated based on the average of all the scores participants got till that point and served as an immediate reward feedback. Participants could know how much money they would eventually earn based on their current level of performance.

The participants were tested in a dark room individually. All displays were presented on a CRT monitor of a 17-inch computer. Participants were instructed to play a game. In this game, they needed to draw circles on the screen by continually pressing the mouse button and dragging the mouse. The center of the circle was the point of the cursor where the button was pressed, and the length of radius was determined by how far the cursor was dragged away from the center point. The circle drawing was completed when participants released the mouse button. Participants would obtain a score ranging from 1 to 100 for each circle they had drawn (Figure 2). Whenever they got “100,” the game ended immediately, and participants got paid the maximum; otherwise, they needed to draw 500 circles and finally got paid according to their average score. At the end of every trial, participants would get a reward feedback, which was based on the average score they obtained till that point. Thus, participants could know how much money they could eventually get according to their current performance. Half of the participants were assigned to the *unknown* condition, and they were not informed any information about the relationship between the circles they drew and the scores they received. The other half of the participants were assigned to the *known* condition and explicitly informed that the scores were randomly generated (i.e., the scores were totally unrelated to the position and size of circles and the way of drawing the circle). The *unknown* condition created an information gap because the participants did not know if there was a relationship between the circle and the score; thus, D-type curiosity was expected to be triggered. Without such uncertainty, in the *known* condition, I-type curiosity would emerge. Before the experiment, each participant would take several trials for practice to eliminate their uncertainties about operation factors. After the game, participants received a questionnaire about their thoughts during the task, for example “please describe your thoughts during this game.” Actually, scores were randomly chosen from 0 to 99. Therefore, “100” never appeared, and each participant needed to draw 500 circles. Five hundred trials were considered to be enough to examine the tendency of exploring behaviors.

In Study 2, drawing time and radii of circles were used to analyze participants’ behaviors. The drawing time could partly reflect the mental efforts that participants expended for drawing circles, for example, thinking about what kinds of circles to draw. The radii intuitively reflected participants’ operation efforts. In the *known* condition, if participants finished the game according to task demands, they should just draw circles without thinking too much. Also, to save time and energy, they could draw circles as small as possible. Thus, both the drawing time and radii of circles would be stable and at a low level. We named this the optimal solution. If participants engaged in unnecessary actions beyond task requirements, the drawing time or radii possibly increased and fluctuated with time. We hypothesized that participants in the *known* condition would engage in explorative activity even when it was unnecessary; while participants in the *unknown* condition would exhibit a stronger tendency to explore. Further, we hypothesized that when they did not reach any conclusion, they would gradually give up exploring and draw circles that were easy to complete (i.e., smaller circles). Thus, the M and SD of both drawing time and size of radii would decrease with the number of trials, and it would take more trials for participants in the *unknown* condition to reach the optimal solution.

### Analysis and Results

**FIGURE 3:**
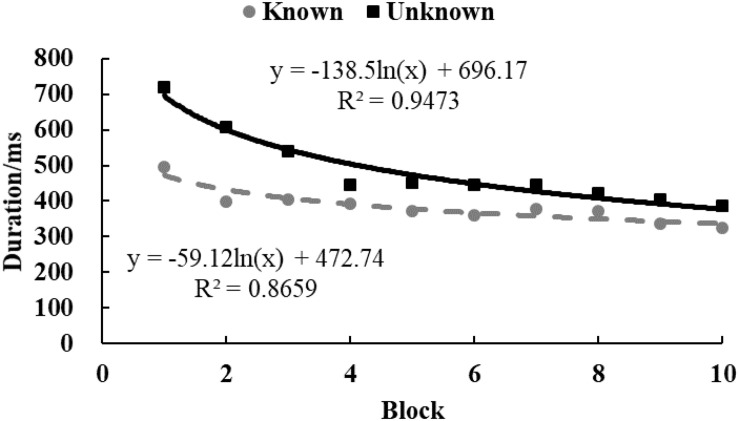
The M of drawing time in Study 2. The solid and dotted lines represent the regression curves for the *unknown* and *known* conditions, respectively.

**FIGURE 4:**
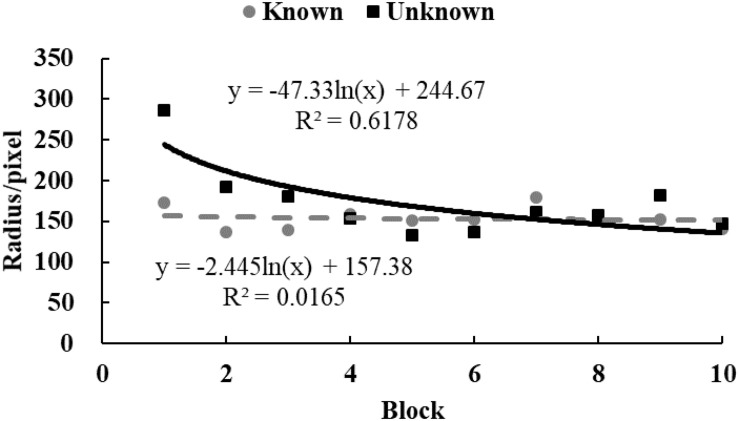
The M of radii size in Study 2. The solid and dotted lines represent the regression curves for the *unknown* and *known* conditions, respectively.

**FIGURE 5:**
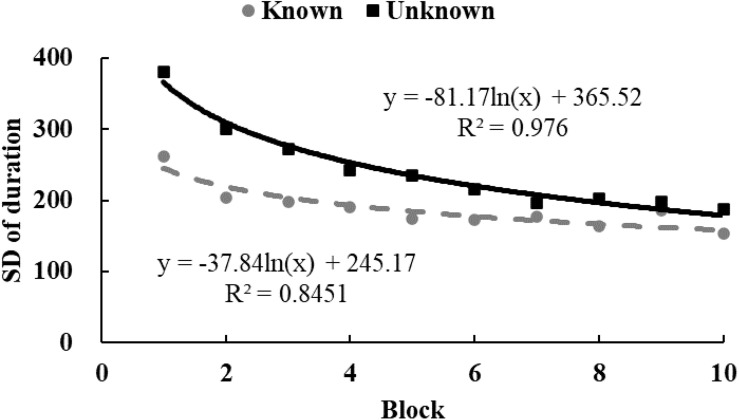
The SD of drawing time in Study 2. The solid and dotted lines represent the regression curves for the *unknown* and *known* conditions, respectively.

**FIGURE 6:**
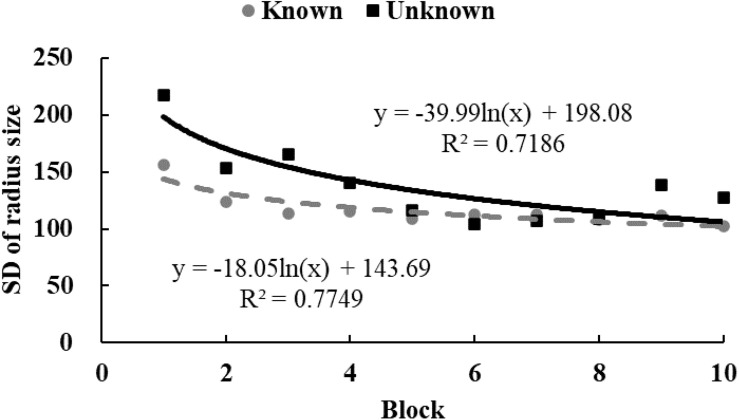
The SD of radii size in Study 2. The solid and dotted lines represent the regression curves for the *unknown* and *known* conditions, respectively.

To examine the tendency of drawing time and radii, data from each participant were equally divided into 10 blocks in chronological order. The M (Figures 3, 4) and standard variance (Figures 5, 6) of drawing time and M radius were calculated for each block. A regression analysis was performed separately for the M and the SD of each dependent variable to reveal the trend of exploration behaviors. Two participants in the *unknown* condition were excluded because their average time used to draw circles lay more than three SDs from the M. Three participants in the *known* condition were excluded owing to the same reason. Therefore, the data from 62 participants (31 in each condition) were analyzed.

In the *unknown* condition, the regression coefficient of the average value for drawing time was significantly different from zero, and the drawing time decreased significantly with blocks [*M* = –138.52, *SD* = 103.86, *t*(30) = 7.43, *p* < 0.001, *d* = 1.33]. In the *known* condition, drawing time also decreased significantly with blocks [*M* = –59.12, *SD* = 139.31, *t*(30) = 2.36, *p* < 0.05, *d* = 0.42]. And as shown in Figure 3, drawing time of participants in the *known* condition got to a stable level sooner than those in the *unknown* condition[*t*(60) = 2.54, *p* < 0.05, *d* = 0.65]. This indicated that participants in the *known* condition gave up exploring sooner. Figure 4 shows that the regression coefficient of radius size for the *unknown* condition is significantly different from zero, but the coefficient in the *known* condition is not significantly different from zero [M_unknown_ = –47.33, SD_unknown_ = 75.11, *t*(30) = 3.45, *p* < 0.01, *d* = 0.63; M_known_ = –2.44, SD_known_ = 45.79, *t*(30) = 0.29, *p* > 0.05, *d* = 0.05]. Furthermore, the regression the coefficient of radius in the *known* condition was significantly larger than the coefficient in the *unknown* condition [*t*(60) = 2.84, *p* < 0.01, *d* = 0.72]. Participants in the *unknown* condition gradually drew smaller circles, and the average size of the circles from participants in the *known* condition remained stable. In both conditions, the regression coefficients of the SD for drawing time [M_unknown_ = –81.17, SD_unknown_ = 64.65, *t*(30) = 6.88, *p* < 0.001, *d* = 1.26; M_known_ = –37.84, SD_known_ = 55.76, *t*(30) = 3.72, *p* < 0.001, *d* = 0.68] and radius [M_unknown_ = –39.99, SD_unknown_ = 53.06, *t*(30) = 4.13, *p* < 0.001, *d* = 0.75; M_known_ = –18.05, SD_known_ = 35.98, *t*(30) = 2.75, *p* < 0.05, *d* = 0.50] were significantly different from zero. The drawing time and size of radius gradually converged with blocks. As shown in Figures 5, 6, participants in the *known* condition tended to approach the stable level sooner than those in the *unknown* condition[SD of duration, *t*(60) = 2.78, *p* < 0.01, *d* = 0.72; SD of radius, *t*(60) = 1.88, *p* = 0.065, *d* = 0.48].

In the *unknown* condition, a questionnaire from one participant was lost and finally 30 valid questionnaires remained. All 30 participants clearly stated that they thought there remained some rules at the beginning and tried different ways to search. Twenty-seven participants became frustrated and gradually gave up. Two participants stuck with exploring until the end, and one participant reported that he/she was in a cycle of giving up and exploration. In the *known* condition, among the 31 participants, twenty-nine participants reported that they wondered about possible rules and tired to explore, and 2 participants just drew circles without thinking. Thirty participants reported they gradually felt frustrated and tried, gave up thinking and just drew circles casually, and 1 participant stuck to exploring until the end.

### Discussion

The M and SD of the drawing time and radius provided different perspectives on the exploring behaviors. When participants gradually gave up exploring, the decrease in mental and physical investment were possibly reflected by the M. It was noted that they spent less time on thinking about how to draw circles, and smaller circles were drawn to save energy. Meanwhile, the SD reflected the shrink of variation in the behavior when participants moderated their acts of exploration. We detected exploration behavior in both conditions, and participants in the *unknown* condition stuck to exploring activities for longer time. The present finding corresponds with former research, which revealed that D-type curiosity trait is associated with more exploration (Litman et al., 2005, 2010; Litman, 2008, 2010; Lauriola et al., 2015). This also indicates that the manipulation to differentiate two types of curiosity was partly valid. We also noted that participants in the *known* condition reported that they tried to understand the possible rules. This statement contradicts the report that they knew that there was no relationship between the scores and circles. Thus, the statement about “seeking rules” is more likely to be a *post hoc* attribution of the superfluous behaviors to account for the fact that they did not know the exact reasons.

Nevertheless, we found no significant decrease in average size of radii for participants in the *known* condition. We assumed that the absence of trend in radii was partly due to the mode of operation in Study 2. In the circle-drawing task, participants were always required to draw a circle. Theoretically, the optimal solution was to draw a circle with a radius approaching zero, however, it was technically difficult for participants to draw such small circles. Thus, they might choose to draw a circle that is normal without thinking too much. Actually, many participants stated that they finally drew circles casually. Moreover, the length of radii recorded were the final consequence of the adjustments, and they did not precisely represent the amount of manual operation. This could also shrink the differences. For example, participants might zoom in and out of the circles repeatedly. However, the radius recorded here only reflects the consequence of modulation but not how many pixels had been changed in total.

Fatigue might be partly responsible for the decline of time. When participants were tired, they naturally spent less time thinking and drew smaller circles. To further test our hypotheses and distinguish from the effect of other factors, we designed Study 3, in which a clear distinction between “doing” and “not doing” was made with more sensitive-dependent variables than Study 2.

## Study 3

Study 3 simulated mobile role-playing games slightly different than Study 2. Rather than drawing circles, participants were informed to assess circles on the screen. They could choose to directly accept circles or to adjust the size and location of circles and then accepted them. Participants would get a score for each circle. If participants adjusted the circles, every pixel they modulated would be recorded. If participants opted not to adjust circles and directly accepted them, the operation amount would be 0 pixels. Therefore, we had an accurate variate to reflect participants’ operation efforts. We also recorded assessment time, which reflected how much time participants used before accepting circles. Other settings were similar with Study 2. We hypothesized that in both conditions, the M and SD of each dependent variable would decrease with time and finally converge at a certain level. Participants in the *unknown* condition would show more exploring activities than participants in the *known* condition. All measures, manipulations, and exclusions in Study 3 are subsequently reported. No more data were collected contingent on initial analysis.

### Materials and Methods

#### Participants

Based on the results of Study 2, we predicted an effect size of Cohen’s *d* = 0.68 for Study 3. Power analysis showed that given α = 0.05, 70 individuals were sufficient to detect an effect with a power of 0.8 (Faul et al., 2007). Seventy participants were finally recruited in this experiment: 34 in the *unknown* condition (25 women; M_age_ = 19.66 years, *SD* = 1.30; age range = 17–23 years) and 36 in the *known* condition (23 women; M_age_ = 20.28 years, *SD* = 1.80; age range = 18–24 years). Participants were paid to participate in the experiment and signed consent forms. All participants had normal or corrected-to-normal visual acuity. The study was approved by the Research Ethics Board at the Department of Psychology and Behavioral Sciences, Zhejiang University.

#### Procedure and Design

**FIGURE 7:**
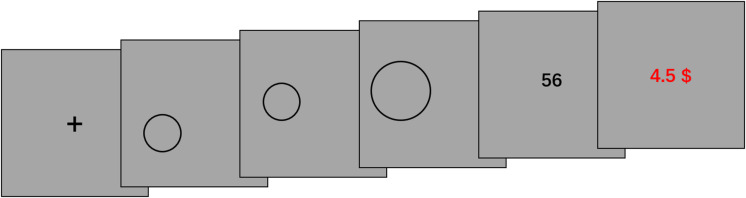
The procedure used in Study 3. For each trial, a fixation cross was presented for 500 ms. Then participants entered the adjustment phase. They could adjust the position and size of the circle by dragging the mouse (the left mouse button for size and the right mouse button for position). Participants pressed the space key to end the adjustment phase. They could also directly press the space key without making any adjustment. After the adjustment phase, the score they received appeared for 1 s. Then, their reward amount appeared on the screen for 750 ms. The reward, which served as an immediate reward feedback, was based on the M of all the scores participants had.

The participants were tested in a dark room individually. All displays were presented on a CRT monitor of a 17-inch computer. In the current game, participants were showed a circle with a fixed radius (close to the average size of the radius in Study 2, 150 pixels) at a random position. They could adjust the position and size of the circle by dragging the mouse (the left mouse button for size and the right mouse button for position). If participants gave up adjusting circles or had finished their adjustment, they could press the space bar to end the adjustment phase. After that, participants received a score ranging from 1 to 100. The *unknown* condition offered an information gap because the participants were uninformed of the relation between scores and circles. Other settings were similar with Study 2. During this game, participants could make no adjustment and just press the space bar to finish the game as soon as possible (Figure 7). The assessing time (from the onset of the circles to pressing the space bar) and amount of operation were analyzed. The operation amount was measured by the total pixels that participants adjusted. For example, if a participant moved a circle 6 pixels rightward, 4 pixels leftward, and 8 pixels downward, the adjustment amount was 18 (6 + 4 + 8) pixels. If they further magnified the radius for 2 pixels and then shrank for 2 pixels, the adjustment amount was 22 (18 + 2 + 2) pixels. Before the experiment, each participant would take several trials for practice to eliminate their uncertainties about operation factors. After the game, participants received a questionnaire about their thoughts during the task, for example, “please describe your thoughts during this game.”

### Analysis and Results

The data analysis methods were exactly the same as those employed in Study 2. There were 34 and 36 participants in the *unknown* and the *known* conditions, respectively. In the *unknown* condition, five were excluded because their assessment time lay more than three SDs from the M, and four were excluded from the *known* condition for the same reason. One participant in the *known* condition was excluded from further analysis because he forgot that there was no relationship between circles and scores. Therefore, the data from 60 participants (29 in the *unknown* condition and 31 in the *known* condition) were analyzed.

**FIGURE 8:**
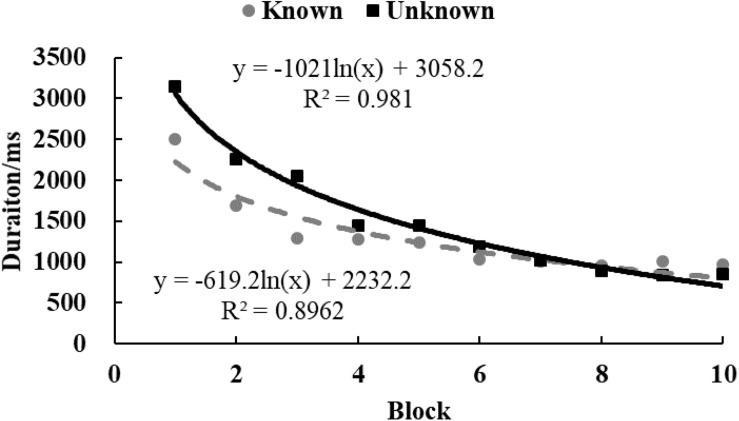
The M of decision time in Study 3. The solid and dotted lines represent the regression curves for the *unknown* and *known* conditions, respectively.

**FIGURE 9:**
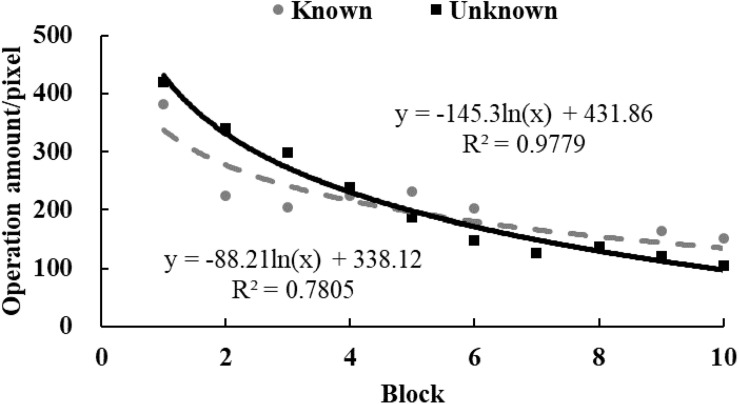
The M of operation amount in Study 3. The solid and dotted lines represent the regression curves for the *unknown* and *known* conditions, respectively.

**FIGURE 10:**
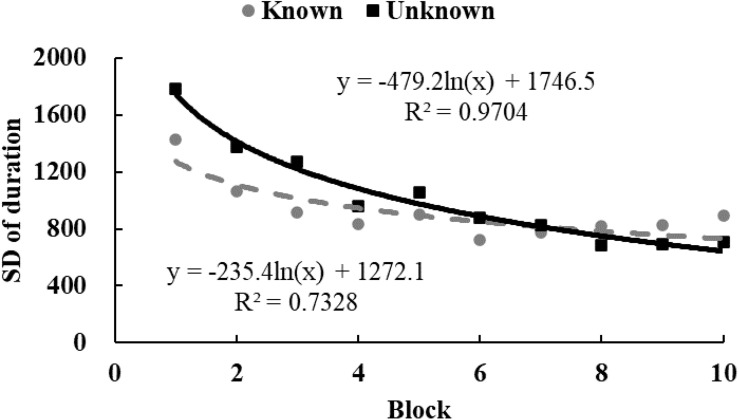
The SD of decision time in Study 3. The solid and dotted lines represent the regression curves for the *unknown* and *known* conditions, respectively.

**FIGURE 11:**
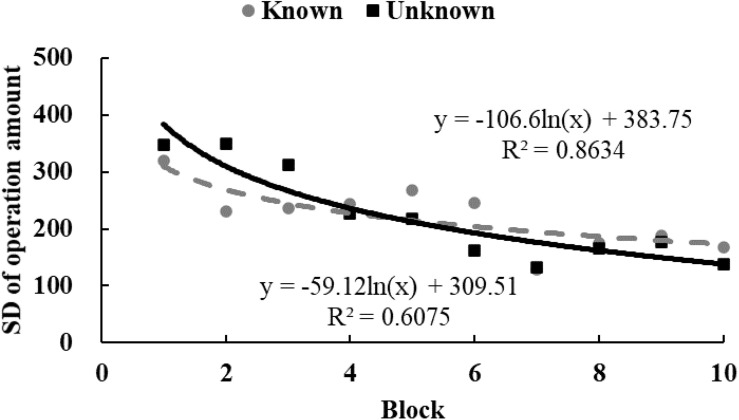
The SD of operation amount in Study 3. The solid and dotted lines represent the regression curves for the *unknown* and *known* conditions, respectively.

In both conditions, the regression coefficient of M of assessing time significantly differed from zero [*M*_unknown_ = –1020.90, SD_unknown_ = 809.54, *t*(28) = 6.79, *p* < 0.001, *d* = 1.26; M_known_ = –618.84, SD_known_ = 589.68, *t*(30) = 5.84, *p* < 0.001, *d* = 1.05]. This indicated that all participants explored at the beginning and gradually gave up, spending less time assessing circles. And as shown in Figure 8, assessing time of participants in the *known* condition reached a lower level sooner than those in the *unknown* condition [*t*(58) = 2.21, *p* < 0.05, *d* = 0.57]. Figure 9 shows that the regression coefficient of operation amount was significantly different from zero in the *unknown* condition [*M* = –145.00, *SD* = 94.98, *t*(28) = 8.22, *p* < 0.001, *d* = 1.53] and in the *known* condition [*M* = –87.95, *SD* = 92.01, *t*(30) = 5.32, *p* < 0.001, *d* = 0.96]. The regression coefficient of operation amount in the *known* condition was significantly larger than the coefficient in the *unknown* condition [*t*(58) = 2.36, *p* < 0.05, *d* = 0.61]. This indicated that participants in the *known* condition started to make no adjustment sooner. In both conditions, the regression coefficients of the SD for assessing time [M_unknown_ = –479.22, SD_unknown_ = 251.65, *t*(28) = 10.08, *p* < 0.001, *d* = 1.90; M_known_ = –235.37, SD_known_ = 315.10, *t*(30) = 4.09, *p* < 0.001, *d* = 0.75] and operation amount [M_unknown_ = –106.64, SD_unknown_ = 95.68, *t*(28) = 5.90, *p* < 0.001, *d* = 1.11; M_known_ = –59.12, SD_known_ = 86.71, *t*(30) = 3.73, *p* < 0.001, *d* = 0.68] were significantly different from zero. This indicated that the variance of exploring activities gradually declined with blocks. As shown in Figures 10, 11, participants in the *known* condition were inclined to converge earlier than participants in the *unknown* condition[SD of assessing time, *t*(58) = 3.24, *p* < 0.01, *d* = 0.86; SD of operation amount, *t*(58) = 1.98, *p* = 0.052, *d* = 0.52].

In the *unknown* condition, 28 participants reported that they were curious and tried to figure out the rules that might exist in Study 3. One participant felt puzzled and gradually bored. Sixteen participants had blocks with no adjustment, and 13 participants adjusted circles in all blocks. In the *known* condition, questionnaires of one participant was lost, and there finally remained 30 valid questionnaires. Twenty-eight participants stated that they wondered about possible rules and tried to explore. The remaining two participants reported that they mainly just pressed the space key. During Study 3, 18 participants had blocks with no adjustment, and 12 participants adjusted circles in all blocks.

### Discussion

In Study 3, we obtained significant exploring behaviors from time and operation amount in both conditions. We also repeated the outcome in previous research (Litman et al., 2005, 2010; Litman, 2008, 2010; Lauriola et al., 2015) that D-type curiosity had a larger impact than I-type curiosity. Participants in the *unknown* condition generally devoted longer time and more physical efforts during the study. In Studies 2 and 3, facing random feedbacks, participants in the *unknown* condition were tougher and kept exploring more than participants in the *known* condition. We inferred that they possibly restrained emotion in order to endure so much random feedback and kept exploring (Lauriola et al., 2015).

The outcome in Study 3 could not be totally interpreted as the effect of fatigue. Fatigue indeed affected exploration behaviors. When individuals were tired, participants had less energy to support the execution of exploration, and they reduced and even stopped exploring for rest. However, explorative activities did not derive from fatigue. Fatigue accumulated during Study 3 only served to reduce the amount of adjustment. Similarly, frustration due to random feedback accounted for the decline instead of the presence of exploration. Hence, we assumed that curiosity played an important role in the GL effect. Fatigue and frustration served to partly reduce the GL effect. In addition, the explorative behaviors at the beginning of Study 3 was less likely caused by boredom. Most participants stated that they felt interested about game at the beginning. Actually, we detected the effect of boredom in later blocks. We found that many participants still manually adjusted circles. They reported to be bored during latter blocks. To dispel boredom, they possibly adjusted circles. Thus, we assumed that boredom could drive the GL effect in later blocks.

## General Discussion

Current studies demonstrated that I-type curiosity could partly induce the GL effect. When no obvious specific uncertainty existed, participants still indulged in unnecessary activities beyond the task requirements. The present research also found that D-type curiosity had a greater impact on exploratory behavior.

### Curiosity and Boredom Account for the GL Effect

The present study detected the effect of both accounts. In the *unknown* condition, when participants did not know how to improve the success rate of docking or get a score of 100, D-type curiosity triggered by the specific uncertainty pushed them to look for rules. In the *known* condition, knowing that exploration did not work in current studies, participants still chose to explore in various ways. This reflected the effect of I-type curiosity. Combining the results of three experiments, we concluded that D-type curiosity could have a greater effect than I-type curiosity. In Study 1, D-type and I-type curiosities both drove most participants to manually control the spaceship. In Studies 2 and 3, D-type curiosity pushed participants in the *unknown* condition to devote more efforts and stick to exploring longer. These results indicated that, in the beginning, the two types of curiosities might have similar influences. Both types of curiosities induced people to begin their exploration. It was seen that I-type curiosity exhibited greater vulnerability toward exploration. When individuals failed to find any interesting new information, they gradually gave up. Meanwhile, as random feedback accumulated, instead of feeling interested, participants felt frustrated and bored. They reduced exploration to a stable level. We assumed that it was boredom that partly prevented participants from completely giving up exploring. It was clearer in Study 3, in which the efforts needed to accomplish the task and to explore were distinguished, and the amount of manual operation did not eventually reach zero. In latter blocks, it seemed that it was boring to just wait till the end. Thus, to dispel boredom, participants chose to do something to arouse themselves, which was similar to taking electric shocks in a previous research (Koerth-Baker, 2016).

Many factors such as goals, self-regulatory strategies, and metacognitive experiences possibly contribute to the findings. D-type curiosity is associated with desire to approach success and avoid failure (Litman, 2008). With such goals, participants are reported to be correlated with emotional restraint and greater thoughtfulness regarding knowledge search (Lauriola et al., 2015). Moreover, the nearer participants thought they were with resolving the uncertainty, the higher intensity of curiosity would be (Loewenstein, 1994; Litman, 2005, 2019; Litman et al., 2005). And when answers are partially retrieved (on the tip of the tongue), D-type curiosity is activated (Litman, 2019). In the *unknown* condition when D-type curiosity was activated, participants’ desires for success were energized (Litman, 2008), and they were inclined to restrain emotion and immerse themselves in the pursuit of goals (Lauriola et al., 2015). Whenever participants figured out possible answers for the specific uncertainty, they might think they were near with the success and triggered more attempts (Litman, 2005, 2019; Litman et al., 2005). Correspondingly, participants in the state of I-type curiosity are associated with desire for new knowledge and fun (Litman, 2005, 2019; Litman et al., 2005; Lauriola et al., 2015). When failing to discover interesting findings, they just gave up. In the future, detailed measurements of those factors are needed to clarify the relations among factors and how they are corresponded with behaviors.

### Implications and Limitations of Current Study

Through the presentation of the GL effect, we do not suggest that humans are irrational or stupid. Exploring the reason for the GL effect improves the comprehension of superfluous behavior. Here we clarify both the positive and negative aspects of the GL effect. We do not know everything about this world. The GL effect actually reserves the chance to break knowledge barriers and obtain novel gains in many domains (Day, 1982; Tomkins and Tway, 1985; Simon, 1992, 2001; Kang et al., 2009; Kashdan and Silvia, 2009; Gruber et al., 2014; Ruan et al., 2015). For instance, Koichi Tanaka received the Nobel Prize in chemistry due to the discovery obtained by his “needless” operation. Meanwhile, unnecessary activities beyond task demand possibly incur waste and loss (Kolko and Kazdin, 1989; Green, 1990; Kruger and Evans, 2009; Hoff and Bashir, 2015; Hsee and Ruan, 2016). We found that after dozens of trials, when participants found no interesting discoveries, they gradually gave up. Thus, in situations where extra manipulation might lead to great loss, thorough knowledge and sufficient training experiences are recommended.

This finding provides some implications to both social theories and practices. On one hand, current research could provide implications for social attribution theories. Classical attribution theories assume that people combine all information to understand others’ behaviors, which are probably attributed to personalities, attitudes, situations, and so on (Kelley and Michela, 1980; Surian et al., 2007; Malle, 2008). However, implicit tendencies (such as the exploration driven by curiosity) are usually neglected in social attributions, leading to misunderstanding of others’ (and even our own) behaviors. Therefore, it is necessary to reveal the detailed mechanism of such implicit tendencies that affect social behavior, to get a better interpretation of the human’s “irrational” behavior. On the other hand, our discovery could afford guidance for social information inquiry (Coenen et al., 2018). To better interact with each other’s, humans often need to dynamically track others’ social information, such as attitudes, traits, and motivations. In such cases, this current study suggests that individuals could focus on effective exploration behaviors and minimize unnecessary impulsive attempts to reduce misapprehension.

The awareness of the knowledge gap is necessary for the feeling of deprivation in the D-type curiosity. The presentation of questions or turned-over pictures spontaneously incur curiosity about the answer of the questions (Litman et al., 2005) or the content of pictures (Hsee and Ruan, 2016). Current research, by contrast, did not present the knowledge gap in such a self-evident way. In the *unknown* condition, participants were aware that there was room for operation. For example, in Study 1, participants could further shrink the deviation when the automatic system finished adjusting; in Studies 2 and 3, participants could adjust the size and location of circles. Meanwhile, the responsibility as astronauts in Study 1 and more awards associated with higher scores in Studies 2 and 3 could encourage participants to improve task performance. Such situations possibly offered the knowledge gap about whether manipulation would improve performance. The knowledge gap generated by the combination of experimental settings was indirect; thus, the effect of D-type curiosity might be underestimated. Further studies with more direct manipulation of the information gap are needed to clarify the differences between D-type and I-type curiosities.

In the present research, we distinguished D-type and I-type curiosities by manipulating the existence of obvious specific uncertainties according to the theoretical differences between two types of curiosities (Loewenstein, 1994; Litman, 2008; Litman et al., 2010; Chang and Shih, 2019). The finding that the D-type state curiosity exerted a stronger effect on energizing exploration behaviors than the I-type state curiosity was consistent with the outcome from research on trait curiosity (Litman et al., 2005). This indicates that current manipulation has differentially involved D-type and I-type curiosities. Considering that the measurement of curiosity states possibly encourages participants to guess the purpose of the research, we did not make direct manipulation checks to avoid potential confounding from high-level cognition. However, the lack of direct measure of curiosity can lead to alternative comprehension of the results, especially in Study 1. Considering the existence of the automatic system, participants possibly controlled the spaceship due to their belief that they could outperform the automatic system (Sieck and Arkes, 2005; Moore and Healy, 2008). Therefore, findings in the current research, particularly in Study 1, could be limited in revealing the peril of curiosity. In the future, systematic measurement of all these factors are needed to further clarify the effect of curiosity and other underlying motives. Trait curiosity measurements are also recommended to further improve the comprehension of the trait-state interactions in exploration.

## Conclusion

Individuals possibly make superfluous efforts just for interest. Three studies revealed participants’ multiple but unnecessary exploration behaviors when specific uncertainty was removed and boredom did not dominate. Those GL effects were partly interpreted as the effect of I-type curiosity. Such superfluous behaviors on one hand leads to waste and loss, and on the other hand also retain the opportunity to obtain novel gains. To make the best use of advantages and bypass the disadvantages of similar “irrational” behaviors, we hope for more efforts on uncovering the veil of them.

## Data Availability Statement

All datasets generated for this study are included in the article/Supplementary Material.

## Ethics Statement

The studies involving human participants were reviewed and approved by the Research Ethics Board of Zhejiang University. The patients/participants provided their written informed consent to participate in this study.

## Author Contributions

JZ, HC, PL, and MS conceived and designed the experiments, performed the data analysis and results interpretation. PL and XL performed the experiments and collected the data, drafted the manuscript. JZ, HC, and MS provided the critical revisions. All authors contributed to the article and approved the submitted version.

## Conflict of Interest

The authors declare that the research was conducted in the absence of any commercial or financial relationships that could be construed as a potential conflict of interest.
